# Rapamycin has a biphasic effect on insulin sensitivity in C2C12 myotubes due to sequential disruption of mTORC1 and mTORC2

**DOI:** 10.3389/fgene.2012.00177

**Published:** 2012-09-11

**Authors:** Lan Ye, Behzad Varamini, Dudley W. Lamming, David M. Sabatini, Joseph A. Baur

**Affiliations:** ^1^Institute for Diabetes, Obesity, and Metabolism, Perelman School of Medicine, University of PennsylvaniaPhiladelphia, PA, USA; ^2^Department of Physiology, Perelman School of Medicine, University of PennsylvaniaPhiladelphia, PA, USA; ^3^Whitehead Institute for Biomedical ResearchCambridge, MA, USA; ^4^Department of Biology, Massachusetts Institute of TechnologyCambridge, MA, USA; ^5^Howard Hughes Medical Institute, Massachusetts Institute of TechnologyCambridge, MA, USA; ^6^Broad Institute of Harvard, Massachusetts Institute of TechnologyCambridge, MA, USA; ^7^The David H. Koch Institute for Integrative Cancer Research at Massachusetts Institute of TechnologyCambridge, MA, USA

**Keywords:** mTOR, mTORC1, rapamycin, mTORC2, insulin sensitivity, mitochondrial biogenesis, chronic, diabetes, raptor, rictor, respiration

## Abstract

Rapamycin, an inhibitor of mTOR complex 1 (mTORC1), improves insulin sensitivity in acute studies *in vitro* and *in vivo* by disrupting a negative feedback loop mediated by S6 kinase. We find that rapamycin has a clear biphasic effect on insulin sensitivity in C2C12 myotubes, with enhanced responsiveness during the first hour that declines to almost complete insulin resistance by 24–48 h. We and others have recently observed that chronic rapamycin treatment induces insulin resistance in rodents, at least in part due to disruption of mTORC2, an mTOR-containing complex that is not acutely sensitive to the drug. Chronic rapamycin treatment may also impair insulin action via the inhibition of mTORC1-dependent mitochondrial biogenesis and activity, which could result in a buildup of lipid intermediates that are known to trigger insulin resistance. We confirmed that rapamycin inhibits expression of PGC-1α, a key mitochondrial transcription factor, and acutely reduces respiration rate in myotubes. However, rapamycin did not stimulate phosphorylation of PKCθ, a central mediator of lipid-induced insulin resistance. Instead, we found dramatic disruption of mTORC2, which coincided with the onset of insulin resistance. Selective inhibition of mTORC1 or mTORC2 by shRNA-mediated knockdown of specific components (Raptor and Rictor, respectively) confirmed that mitochondrial effects of rapamycin are mTORC1-dependent, whereas insulin resistance was recapitulated only by knockdown of mTORC2. Thus, mTORC2 disruption, rather than inhibition of mitochondria, causes insulin resistance in rapamycin-treated myotubes, and this system may serve as a useful model to understand the effects of rapamycin on mTOR signaling *in vivo*.

## INTRODUCTION

Rapamycin is the only drug that has been unequivocally established to robustly and reproducibly extend maximum lifespan in mice (Harrison et al., 2009). However, very little is known about the changes in physiology or intracellular signaling that accompany this effect. Interestingly, rapamycin was tested for effects on lifespan in part because it is proposed to mimic some of the signaling events triggered by caloric restriction (CR), which extends life in most species that have been tested (Weindruch and Walford, 1988; Baur, 2009; Cox and Mattison, 2009). Two hallmarks of CR in rodents are improved insulin sensitivity (McKee Alderman et al., 2010) and increased mitochondrial biogenesis (Nisoli et al., 2005). Surprisingly, rapamycin causes insulin resistance (Houde et al., 2010; Lamming et al., 2012) and inhibits both the production and function of mitochondria, at least in cells (Schieke et al., 2006; Cunningham et al., 2007; Ramanathan and Schreiber, 2009). It is therefore clear that rapamycin does more than act as a simple “CR mimetic,” and understanding the molecular mechanisms involved has the potential to lead to both the development of more effective molecules, and new insights into the underlying processes that regulate mammalian aging.

The canonical target of rapamycin is mechanistic (formerly mammalian) target of rapamycin complex 1 (mTORC1), a kinase involved in nutrient sensing and the regulation of growth and metabolism (Sabatini et al., 1994). The mTORC1 pathway is suppressed by CR in a variety of mammalian tissues (e.g., Estep et al., 2009; Lashinger et al., 2011; Shinmura et al., 2011), and interfering with this pathway genetically in yeast results in lifespan extension that is not additive with CR in that organism (Kaeberlein et al., 2005). Although complete elimination of mTORC1 in mammals causes embryonic lethality (Guertin et al., 2006), animals lacking one of its substrates, S6K1, are long-lived and display enhanced insulin sensitivity (Selman et al., 2009), supporting the idea that important features of CR may be mediated by suppression of this pathway. We have recently described a strain of mice with mildly impaired mTORC1 signaling that are also long-lived (Lamming et al., 2012). However, these mice lack the enhanced insulin sensitivity that is conferred by total S6K1 ablation, suggesting that the two effects are separable. Consistent with this, we and others have reported that rodents treated with rapamycin are overtly insulin resistant (Houde et al., 2010; Lamming et al., 2012), despite the longer lifespan of rapamycin-treated mice (Chen et al., 2009; Harrison et al., 2009).

**FIGURE 1:**
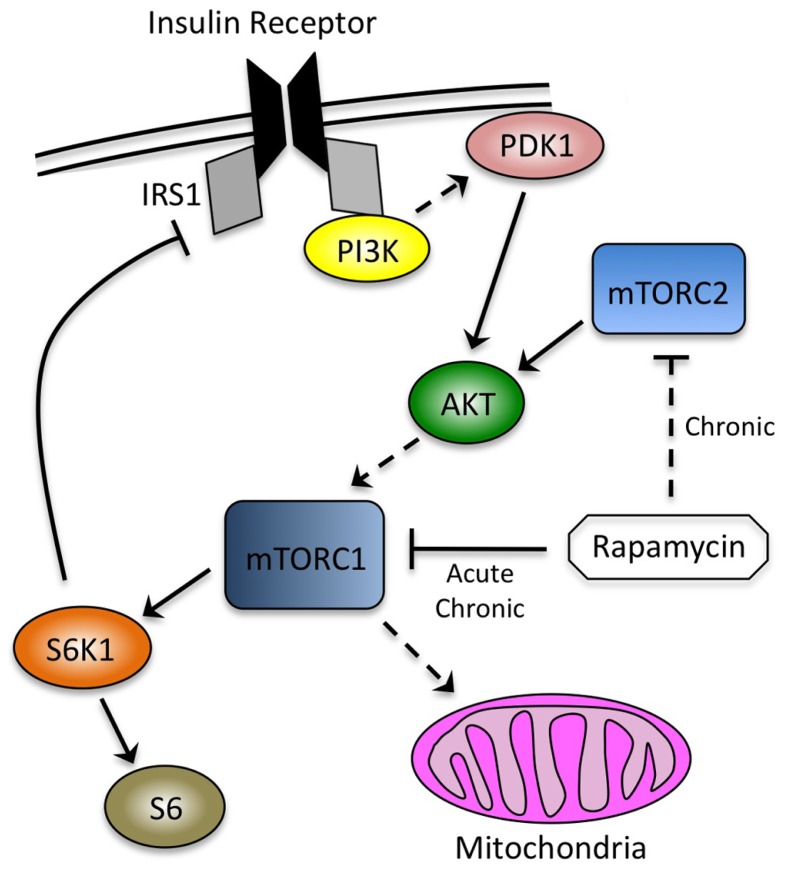
**Interdependence of signaling through the insulin receptor and mTOR.** mTORC1, the canonical target of rapamycin, is downstream of the insulin signaling cascade and mediates negative feedback through S6K1. mTORC2 is an AKT kinase that is not sensitive to acute rapamycin treatment (~ h), but can be inhibited by chronic exposure (~24 h). Phosphorylation of AKT by PDK1 and mTORC2 occurs at discrete sites (threonine 308 and serine 473, respectively). mTORC1 promotes mitochondrial biogenesis, although the relevance of this observation to its effects on insulin sensitivity has not been established. Solid lines indicate direct effects, whereas dashed lines indicate intermediate steps, or in the case of rapamycin inhibiting mTORC2, the requirement for chronic exposure.

We have shown that rapamycin-induced insulin resistance in mice is at least partially due to disruption of mTORC2, a second mTOR-containing complex that phosphorylates several substrates, including AKT at serine 473 as part of the insulin signaling cascade (**Figure 1**; Lamming et al., 2012). mTORC2 is not acutely sensitive to rapamycin, but was shown to be disrupted in some, but not all, cultured cell lines after continuous exposure to the drug (Sarbassov et al., 2006). In addition, loss of AKT phosphorylation at serine 473 after prolonged rapamycin treatment has been observed *in vivo*, consistent with our biochemical demonstration that mTORC2 can be disrupted in intact tissues in mice.

A possibility that has not been adequately addressed to date is that decreased abundance or function of mitochondria could contribute to the detrimental effects of rapamycin on metabolism. In stark contrast to CR (Nisoli et al., 2005), rapamycin has been shown to suppress mitochondrial biogenesis (Cunningham et al., 2007), and to acutely decrease mitochondrial respiration in cultured cells (Schieke et al., 2006; Ramanathan and Schreiber, 2009). A large body of evidence supports the contention that insufficient mitochondrial capacity in skeletal muscle can trigger a buildup of lipid-derived molecules, including diacylglycerols and ceramides, that subsequently trigger activation of PKCθ, inhibitory serine phosphorylation of IRS1, and insulin resistance (Morino et al., 2006; Erion and Shulman, 2010).

In C2C12 myotubes, we have observed a clear biphasic effect of rapamycin on insulin sensitivity. We show that an early (~ h) improvement in insulin responsiveness correlates to the acute loss of signaling through S6K1, consistent with disruption of the negative feedback loop driving inhibitory phosphorylation of IRS1 (**Figure 1**; Um et al., 2004). At later time points (>24 h), however, we find that myotubes become refractory to the effects of insulin. We provide biochemical and genetic evidence to show that disruption of mTORC2, rather than mitochondrial dysfunction, plays the dominant role in rapamycin-induced insulin resistance. These studies expand our understanding of the molecular consequences of rapamycin exposure, and how these changes influence insulin sensitivity in a cell culture model of skeletal muscle.

## MATERIALS AND METHODS

### MATERIALS

Antibodies to phospho-AKT S473 (9271), phospho-AKT T308 (9275), AKT (9272), phospho-S6 ribosomal protein (2215), S6 ribosomal protein (2217), mTOR (2972), Raptor (2280), Rictor (2140), phospho-PKC theta T538 (9377), and VDAC (4866) were from Cell Signaling Technology. The phospho-IRS1 S307 (07-247) antibody was from Upstate and IRS1 (05-1085) and GAPDH (JC1641540) antibodies were from Millipore. OXPHOS cocktail (MS604/D1848) was from MitoSciences. Protease and phosphatase inhibitor cocktail tablets were from Roche (11836153001 and 04906845001, respectively). Rapamycin was purchased from Calbiochem (553210). Torin1 was a gift from the lab of Nathanael Gray. Triglyceride reagent was from Stanbio Laboratory. DMEM, fetal bovine serum (FBS), horse serum, insulin, protein A agarose, and Trizol were obtained from Invitrogen. Other chemicals were purchased from Sigma unless noted.

### CELL CULTURE

C2C12 mouse myoblasts were maintained in DMEM supplemented with 10% FBS and antibiotics (Pen/Strep). When cells reached confluence, the medium was switched to DMEM with 2% horse serum. Cells were refreshed with differentiation medium every other day. By day 4, C2C12s were fused into myotubes. C2C12 myotubes were then treated with 500 nM rapamycin for the indicated times. Torin1 treatment was at 250 nM for 4 h. Palmitate-containing media were prepared by conjugating palmitate with FFA-free bovine serum albumin as described by Chavez et al. (2005). Briefly, palmitate was dissolved in ethanol (75 mM), diluted 1:25 in DMEM containing 2% BSA, sonicated briefly, and incubated at 55^°^C for 10 min. The complexed palmitate was then diluted to a final concentration of 0.5 mM in DMEM containing 2% BSA, cooled at RT, filtered and administrated to C2C12 myotubes for 16 h.

### OXYGEN CONSUMPTION RATE MEASUREMENT

C2C12 myoblasts were seeded at 40,000 cells per well in 24-well XF plates. Myoblasts were treated with rapamycin for the indicated times, or alternatively, myotubes were generated by replacing the medium with differentiation medium when cells reached confluence. After an 4 days of differentiation, myotubes were treated with rapamycin for the indicated times. The cells were refreshed with non-buffered pH 7.4 medium (25 mM glucose) and incubated for 1 h in a non-CO_2_ incubator, as recommended by Seahorse Biosciences. A Seahorse XF24 Analyzer (Seahorse Biosciences) was then used to measure the oxygen consumption rate. Each cycle included 4 min of mixing, a 2-min wait, and then measurement over 2 min. One micromolar oligomycin was used to inhibit ATP synthase. Next, 300 nM FCCP was added to stimulate uncoupled respiration. Immediately after measurement, the protein concentration was measured by bicinchoninic acid (BCA) assay (Pierce Biotechnology).

### WESTERN BLOTTING

Cells were rinsed with PBS and lysed in cold RIPA buffer supplemented with phosphatase inhibitor and protease inhibitor cocktail tablets. Cell lysates were incubated on ice for 10 min, sonicated on ice for 30 s, and centrifuged at 12,800 rpm for 15 min at 4^°^C. Protein concentration was determined by BCA assay (Pierce Biotechnology). Twenty microgram proteins were separated by sodium dodecyl sulfate-polyacrylamide gel electrophoresis (SDS-PAGE) on 8–16% gradient or 7.5% resolving gels. Quantification was performed by densitometry using ImageJ software, and loading was verified by blotting for GAPDH.

### IMMUNOPRECIPITATIONS

C2C12 myotubes were lysed in cold 0.3% CHAPS lysis buffer [40 mM Hepes (pH 7.5), 120 mM NaCl, 1 mM EDTA, 0.3% CHAPS, 10 mM pyrophosphate, 10 mM β-glycerophosphate, 50 mM NaF, 0.5 mM orthovanadate, and protease inhibitors]. Cells lysates were incubated at 4^°^C for 15 min and then centrifuged at 16,000 rpm for 15 min at 4^°^C to remove insoluble material. Protein A agarose beads were added to the supernatant and incubated with rotation for 1 h. Next, the beads were centrifuged out of the lysates and mTOR or Rictor antibodies were added to the cleared lysates, which were rotated overnight at 4^°^C. Protein A agarose beads were added to the supernatant and incubated at 4^°^C for an additional hour. Immunoprecipitated complexes with protein A agarose beads were washed in 0.3% CHAPS lysis buffer three times, boiled in SDS-sample buffer, separated by SDS-PAGE (7.5% gels), and analyzed by immunoblotting. This method is adapted from that described by Sarbassov et al. (2006).

### QUANTITATIVE REAL TIME RT-PCR ASSAY

Total RNA was extracted from the sample using TRIzol reagent. The concentration and purity of RNA were determined by absorbance at 260/280 nm. One microgram of total RNA was reverse transcribed using a high-capacity cDNA reverse transcription kit (Applied Biosystems) according to the manufacturer’s instructions. The cDNA was subjected to real time PCR using SYBR Q-PCR master mix (Applied Biosystems). Primer sequences used to produce gene-specific amplicons are as follows: PGC-1α: forward: ACTATGAATCAAGCCACTACAGAC; reverse: TTCATCCCTCTTGAGCCTTTCG, GAPDH: forward: GGTGAAGGTCGGAGTCAACGGA; reverse: GAGGGATCTCG- CTCCTGGAAGA, TFAM: forward: AAGACCTCGTTCAGCATA- TAACATT; reverse: TTTTCCAAGCCTCATTTACAAGC, A typical reaction contained 250 nM of forward and reverse primer, 1 μl cDNA and the final reaction volume was 20 μl. The reaction was initiated by preheating at 50°C for 2 min, followed by 95°C for 10 min. Subsequently, 40 amplification cycles were carried out with 15 s denaturation at 95°C and 30 s annealing and extension at 60°C. Gene expression was normalized to GAPDH.

### mtDNA DETERMINATION

Briefly, cell pellets were digested in with 15 μl proteinase K (10 mg/ml) in a 500 μl total volume of proteinase K buffer (100 mM Tris-HCl pH 8.5, 5 mM EDTA, 0.2% SDS, 200 mM NaCl) overnight at 55^°^C. 170 μl of 5 M NaCl was added and samples were mixed for 1 min and then centrifuged at max speed for 15 min at 4^°^C. Supernatants were collected and 1 ml ethanol was added, after which the tubes were inverted several times. Samples were centrifuged at max speed for 15 min at 4^°^C, after which ethanol supernatant was aspirated and DNA pellet was washed in 500 μl of 70% ethanol. Samples were centrifuged for 5 min, max speed at 4^°^C, ethanol supernatant was removed, the DNA pellet was air dried and resuspended in 50 μl of TE. Primer sequences used to produce mitochondrial and nuclear specific DNA products for quantification of mtDNA/nuclear DNA ratios are as follows: MT-CO1: forward: TGCTAGCCGCAGGCATTAC; reverse: GGGTGCCCAAAGAATCAGAAC, Ndufv1: forward: CTTCCCCACTGGCCTCAAG; reverse: CCAAAACCCAGTGATCCAGC.

### KNOCKDOWN OF RICTOR AND RAPTOR WITH LENTIVIRUS

293T cells were from ATCC and were seeded onto 10 cm diameter dishes with DMEM (supplemented with 10% FBS and penicillin–streptomycin) and incubated overnight. When the cells became 70% confluent, they were refreshed the cells with antibiotic-free DMEM. Next, the Rictor shRNA, Raptor shRNA, and control GFP shRNA plasmids (Guertin et al., 2009; Thoreen et al., 2009), together with helper vectors pCMV-dR8.2dvpr and pCMV-VSVG, were cotransfected into 293T cells using Fugene reagent. Media containing lentivirus were harvested after 48 h and passed through a 0.4 μM filter. C2C12 myoblasts were infected with lentivirus-containing media for 24 h and selected with 2 μg/ml puromycin. The cell lysates were harvested at appropriate time points and analyzed by immunoblotting.

### MEASUREMENT OF TRIGLYCERIDES

C2C12 myotubes were lysed in PBS containing 1% Nonidet P-40. Triglycerides were determined by triglyceride reagent according to manufacture’s instructions (Stanbio Laboratory). Intracellular triglycerides were normalized to total protein content.

### STATISTICAL ANALYSIS

**FIGURE 2:**
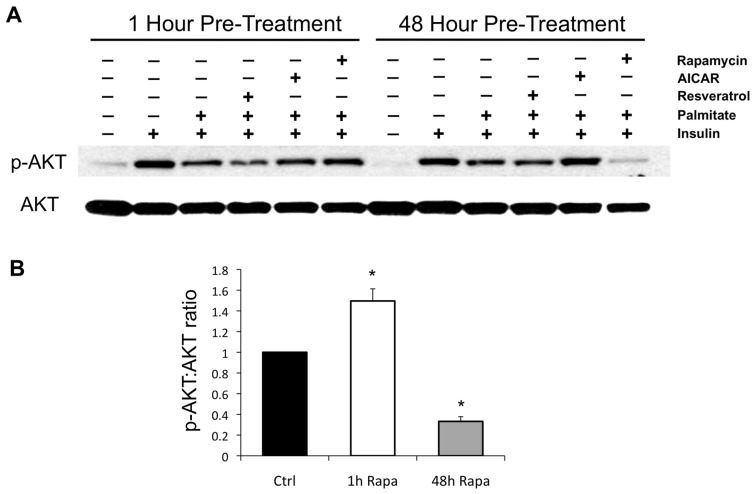
**Effects of rapamycin, AICAR, and resveratrol on palmitate-induced insulin resistance. (A)** Palmitate renders C2C12 myotubes refractory to insulin stimulation, as reflected by downstream phosphorylation of AKT. One hour of pre-treatment with resveratrol mildly increases insulin resistance, consistent with previous reports of acute inhibition of insulin signaling (Zhang, 2006), whereas the AMPK activator AICAR is without effect, and rapamycin slightly improves insulin signaling, likely due to disruption of negative feedback through S6K1 (Um et al., 2004). After 48 h of pre-treatment, AICAR rescues insulin signaling, while rapamycin renders myotubes almost completely resistant. The effects of AICAR and rapamycin on insulin signaling were each verified in at least three independent experiments and representative blots are shown. **(B)** Quantification of the effects of short- (1 h) and long-term (48 h) exposure to rapamycin on the insulin-stimulated phosphorylation of AKT in palmitate-treated C2C12 myotubes. *p < 0.05. Error bars show SEM.

Densitometry was performed using ImageJ software (NIH, Bethesda, MD) and band intensities were normalized to GAPDH, unless otherwise indicated. Significance was tested using the two-tailed unpaired Student’s *t*-test in Microsoft Excel (Microsoft, Seattle, Washington) or a one-sample *t*-test to test whether ratios of phosphorylated Akt differed significantly from the control value (set to one) in **Figures 2 and 6**. * denotes *p* < 0.05 compared to control unless otherwise indicated.

## RESULTS

We initially chose to explore the effects of rapamycin in differentiated C2C12 myotubes cultured in the presence of palmitate because it is known that insulin sensitivity in this system is decreased by the accumulation of lipid intermediates, such as diacylglycerols and ceramides, and that enhancing mitochondrial fatty acid oxidation is sufficient to improve insulin sensitivity (Coll et al., 2010). Therefore, we expected that any major influence of rapamycin on mitochondrial function would be reflected as a change in insulin signaling to AKT.

We found that pre-treatment of C2C12 myotubes with rapamycin for 1 h caused a small, but reproducible improvement in insulin sensitivity, likely due to the loss of negative feedback through S6K1, a substrate of the canonical rapamycin target, mTORC1 (**Figure 2**). Over four independent experiments, the average effect of short-term rapamycin was an approximately 50% increase in the pAkt:Akt ratio following insulin stimulation, *p* = 0.02. Interestingly, when the rapamycin pre-treatment was extended to 48 h, the myotubes became almost completely unresponsive to insulin stimulation, consistent either with further antagonism of lipid-induced insulin resistance via inhibition of mitochondria, or with disruption of mTORC2. Over four independent experiments, the average effect was a 67% decrease in the pAkt:Akt ratio, *p* < 0.001. AICAR, an AMPK activator that promotes respiration and mitochondrial biogenesis (Ojuka, 2004), had the opposite effect, improving insulin sensitivity at the 48 h time point, whereas a second compound known to promote mitochondrial biogenesis, resveratrol, had no effect.

**FIGURE 3:**
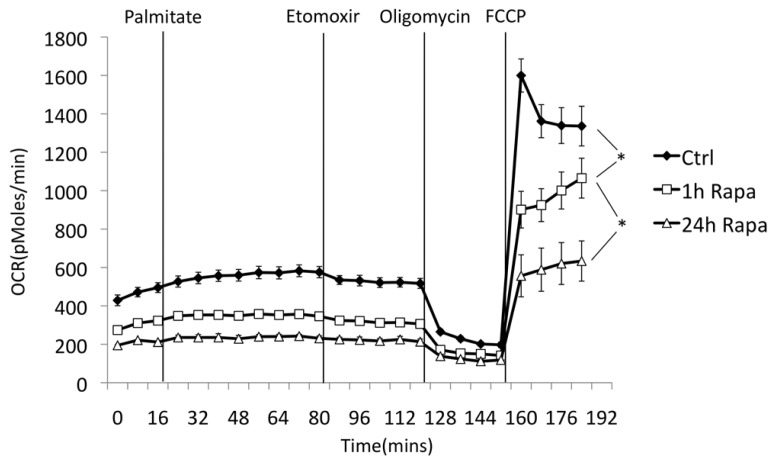
**Rapamycin suppresses oxygen consumption in myotubes.** Oxygen consumption was measured using a Seahorse XF24 Flux Analyzer. C2C12 myoblasts were differentiated on Seahorse plates and pre-treated with rapamycin or vehicle (DMSO) for the indicated times. Glucose was maintained at 25 mM throughout the experiment. Acute addition of palmitate (0.5 mM final concentration, in complex with BSA) had only a minor effect on total oxygen consumption, which was reversed with etomoxir (50 μM), an inhibitor of carnitine palmitoyl transferase 1. Oligomycin was then added to inhibit ATP synthase and determine the rate of proton leak across the mitochondrial membrane. Finally, FCCP was added to uncouple mitochondria and measure maximal respiration rate. Decreased respiration in rapamycin-treated cells was verified in three independent experiments, consistent with previous reports (Schieke et al., 2006; Ramanathan and Schreiber, 2009). The length of rapamycin pre-treatment (1 h vs. 24 h) had a significant effect on respiration rate under basal, but not uncoupled conditions. *p < 0.05. Error bars show SEM.

To test whether rapamycin was sufficient to suppress mitochondrial function, we measured respiration rates in treated or untreated myotubes using a Seahorse XF24 Flux Analyzer. As shown in **Figure 3**, pre-treatment with rapamycin caused a significant reduction in respiration rates under basal, oligomycin-inhibited (reflecting proton leak), and uncoupled conditions (all in the presence of 25 mM glucose). To test whether rapamycin might have changed the substrate preference of the mitochondria, we also added palmitate to the media. However, the addition of a fatty acid substrate did not restore respiration rates in the rapamycin-treated cells, and had only a minor effect on oxygen consumption, which was reversed upon addition of the carnitine palmitoyl transferase 1 (CPT-1) inhibitor etomoxir. Intriguingly, a significant reduction in oxygen consumption occurred after 1 h of pre-treatment, suggesting that it was largely due to changes in flux through the existing mitochondria, rather than to a reduction in the rate of mitochondrial biogenesis.

**FIGURE 4:**
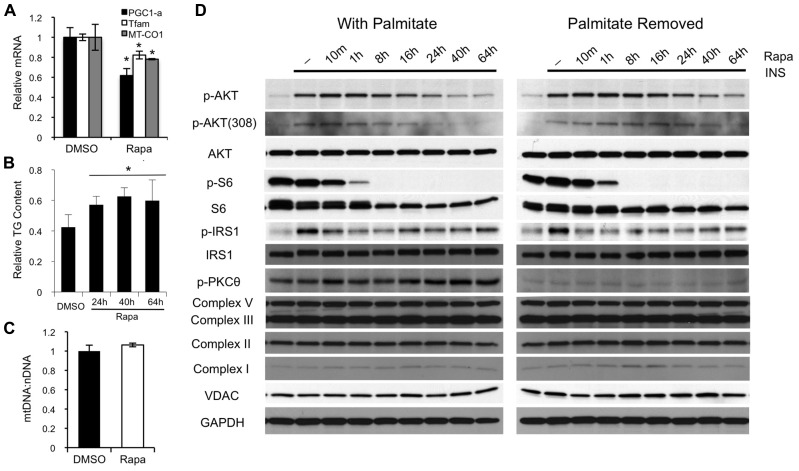
**Rapamycin inhibits mitochondrial transcription factors, but has no major effect on mitochondrial protein levels or PKCθ phosphorylation. (A)** Forty-eight hours of rapamycin treatment decreases expression of PGC-1α and TFAM, two of the major transcription factors involved in mitochondrial biogenesis, as well as the level of MT-CO1, an mtDNA-encoded transcript. **(B)** Rapamycin mildly increases intracellular triglyceride levels in myotubes. The result show is representative of three separate experiments. **(C)** Forty-eight hours of rapamycin treatment has no effect on mitochondrial DNA copy number, as reflected by the ratio between MT-CO1 (mitochondrial DNA-encoded) and Ndufv1 (nuclear-encoded) DNA. **(D)** Preincubation of myotubes with rapamycin for 24 h or more induces insulin resistance irrespective of the presence of palmitate. Rapamycin-induced insulin resistance is not associated with major increases in serine phosphorylation of IRS1 or phosphorylation of PKCθ, two hallmarks of insulin resistance caused by insufficient oxidation of fatty acids. Moreover, there is no change in the expression of several proteins involved in electron transport complexes or in the level of VDAC, a structural protein in the mitochondria. *p < 0.05. Error bars show SEM.

Next, we examined factors related to mitochondrial biogenesis and lipid-induced insulin resistance in rapamycin-treated myotubes. Although we confirmed the previously reported suppression of mitochondrial transcription factors (PGC-1α and TFAM; **Figure 4A**; Cunningham et al., 2007) and were able to show a small increase in triglyceride accumulation (**Figure 4B**), we did not detect any significant effect of rapamycin on mitochondrial DNA content or the levels of various mitochondrial proteins over the time course of this experiment (**Figures 4C,D**). Lipid-induced insulin resistance is typically associated with increased phosphorylation of atypical PKC isoforms and inhibitory (serine) phosphorylation of IRS1. Serine phosphorylation of IRS1 was not enhanced. In fact, IRS1 phosphorylation was diminished in the presence of rapamycin at all time points studied, likely reflecting the inhibition of S6K1. Phosphorylated (active) PKCθ (also called PRKCQ), was clearly higher in the presence of palmitate, and appeared to be incrementally increased by rapamycin. However, there was no induction of PKCθ phosphorylation by rapamycin in the absence of palmitate, yet the myotubes still became insulin resistant (**Figure 4D**). Together, these observations strongly suggested that mitochondrial dysfunction, leading to lipid-induced insulin resistance is not the major mechanism accounting for reduced insulin sensitivity in rapamycin-treated myotubes.

**FIGURE 5:**
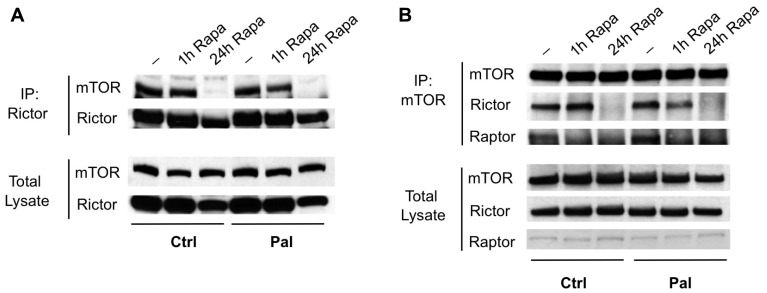
**Rapamycin disrupts mTORC2 in myotubes.** mTORC2 was immunoprecipitated from myotubes that were pre-treated with vehicle, rapamycin for 1 h, or rapamycin for 24 h, using antibodies to Rictor **(A)** or mTOR **(B)**. The integrity of the complex was assessed by blotting for the opposite subunit. In each case, mTORC2 formation was disrupted at 24 h despite only minor changes in total protein levels. The mTORC1 subunit Raptor was also absent from mTOR immunoprecipitates at either 1 or 24 h.

Next, we assessed the integrity of the mTORC2 complex. We found that 24 h, but not 1 h, of rapamycin exposure is sufficient to almost completely disrupt mTORC2 in C2C12 myotubes, as assessed by immunoprecipitation of either Rictor (an mTORC2-specific component) or the mTOR catalytic subunit (**Figure 5**). These results could account for the loss of AKT phosphorylation at serine 473, which appears to be required for subsequent phosphorylation by PDK1 at threonine 308 in many cases (Sarbassov et al., 2005). However, it has been reported that loss of mTORC2 in MEFs or in skeletal muscle selectively impairs serine 473 phosphorylation, with unchanged or even enhanced phosphorylation of threonine 308 (Jacinto et al., 2006; Bentzinger et al., 2008). We therefore wondered whether the loss of threonine 308 phosphorylation in our myotubes was indicative of a second mechanism contributing to insulin resistance, or simply a consequence of mTORC2 loss.

**FIGURE 6:**
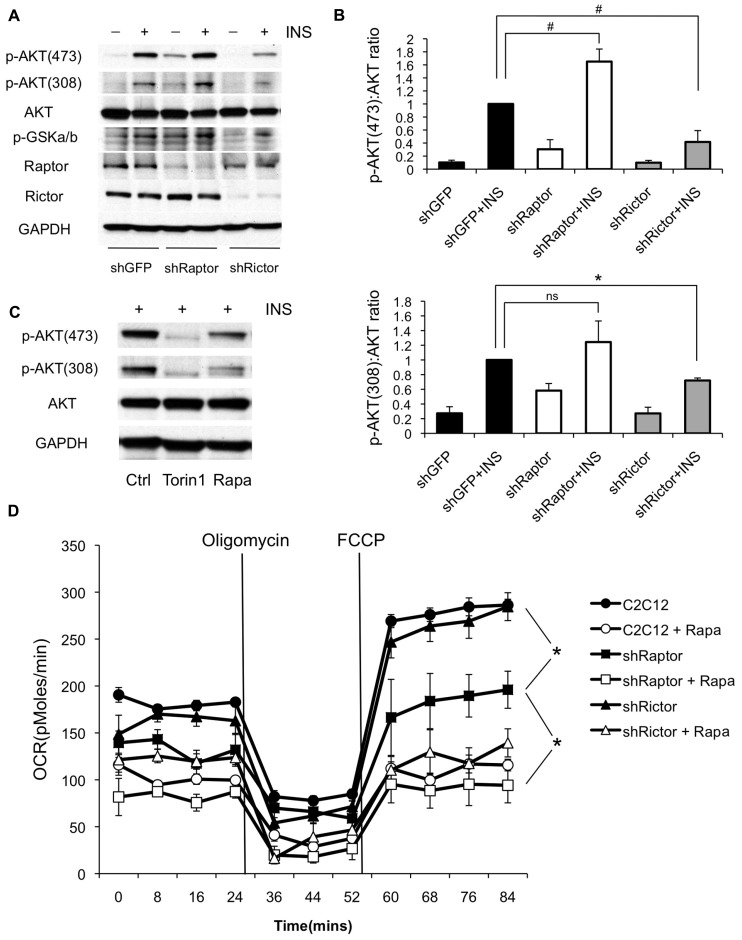
**Knockdown of mTORC1 reduces oxygen consumption while knockdown of mTORC2 causes insulin resistance in myotubes. (A)** C2C12 myoblasts were infected with shRNAs targeting Raptor (mTORC1) or Rictor (mTORC2) and differentiated. Cells were harvested with or without insulin stimulation and probed for phosphorylation of AKT at threonine 308 and serine 473. Results shown are representative of three independent experiments. **(B)** Quantification of changes in the ratio of phosphorylated to total AKT following knockdown of mTOR complex subunits. **(C)** Torin1, an inhibitor of both mTORC1 and mTORC2 is sufficient to block phosphorylation of AKT at both sites, although direct inhibition of upstream phosphoinositide 3-kinase may contribute to this effect (Liu et al., 2012). C2C12 myoblasts were differentiated and treated with Torin1 (250 nM for 4 h) or rapamycin (500 nM for 24 h) **(D)** Knockdown of Raptor partially suppresses oxygen consumption in the absence of rapamycin. C2C12 myoblasts were pre-treated with rapamycin or vehicle (DMSO) for 24 h and then analyzed on the Seahorse XF24 Flux Analyzer in unbuffered DMEM (25 mM glucose) in the presence of rapamycin or DMSO, as indicated. Oligomycin was added to inhibit ATP synthase in order to determine the rate of proton leak across the mitochondrial membrane, and FCCP was subsequently added to uncouple mitochondria and measure maximal respiration rate. ^#^*p* < 0.05 one-tailed; *p < 0.05 two-tailed; ns, not significant. Error bars show SEM.

To confirm the specificity of rapamycin’s effects, we used shRNAs to selectively ablate either mTORC1 or mTORC2 by targeting the complex-specific components Raptor or Rictor, respectively (Guertin et al., 2009; Thoreen et al., 2009). As expected, Raptor knockdown recapitulated both the improved insulin sensitivity due to loss of negative feedback through S6K1, and the inhibition of respiration seen in rapamycin-treated cells (**Figure 6**). On the other hand, Rictor knockdown significantly diminished AKT phosphorylation at serine 473 as well as threonine 308, confirming that this site is sensitive to mTORC2 inhibition in C2C12 myotubes (**Figure 6A**). Torin1, a direct inhibitor of both mTORC1 and mTORC2 (Thoreen et al., 2009), also blocked AKT phosphorylation at both threonine 308 and serine 473 (**Figure 6B**), although it should be noted that inhibition of upstream signaling through PI3K may have contributed to the Torin1 result (Liu et al., 2012). Taken together, our results demonstrate that rapamycin-induced insulin resistance in this system is due primarily to mTORC2 disruption, rather than to mitochondrial effects of the drug.

## DISCUSSION

We have shown that both the short-term improvement in insulin sensitivity and the longer-term loss of insulin sensitivity that are caused by rapamycin *in vivo* can be mimicked in C2C12 myotubes, and that this system can be used to explore the underlying molecular mechanisms.

We chose C2C12 myotubes as a cell culture model to test the contribution of mitochondria to rapamycin-induced insulin resistance for a number of reasons. First, insulin resistance is readily induced in this model with palmitate, and can be ameliorated using a small molecule that enhances mitochondrial fatty acid oxidation (Coll et al., 2010). Second, the ability of rapamycin to inhibit mitochondrial biogenesis was first demonstrated in C2C12 myotubes, and has only been thoroughly characterized in this system (Cunningham et al., 2007). Third, we have shown that AKT phosphorylation is robustly reduced *in vivo* in skeletal muscle by rapamycin treatment (Lamming et al., 2012). The effect of chronic rapamycin on mTORC2 complex formation was originally explored in a series of immortalized and tumor cell lines, many of which proved refractory to the drug for reasons that remain unclear (Sarbassov et al., 2006). Herein, we show that this differentiated, post-mitotic cell type remains sensitive to mTORC2 disruption by rapamycin.

Rapamycin has previously been established to inhibit both mitochondrial biogenesis (Cunningham et al., 2007) and oxidative phosphorylation by existing mitochondria (Schieke et al., 2006; Ramanathan and Schreiber, 2009), raising the possibility that a buildup of lipid intermediates due to insufficient fatty acid oxidation might trigger insulin resistance through the PKCθ to IRS1 signaling cascade in rapamycin-treated cells (Morino et al., 2006). Indeed, palmitate-induced insulin resistance correlated with a pronounced induction of PKCθ phosphorylation that was mildly augmented by rapamycin. Rapamycin also caused a decrease in the expression of key mitochondrial transcription factors and an acute suppression of respiration in our differentiated C2C12 myotubes. However, we found that mTORC2 disruption, rather than mitochondrial dysfunction, was the major cause of insulin resistance after chronic rapamycin exposure.

Nevertheless, the incremental increase in triglyceride buildup, increase in PKCθ phosphorylation in palmitate-treated cells, and reduced expression of PGC-1α and TFAM, hint that mitochondrial effects might exacerbate the insulin resistance caused by mTORC2 disruption, particularly in longer-term experiments *in vivo*. The lack of an appreciable decrease in mitochondrial proteins over the course of 64 h is perhaps not surprising, since many have half lives on the order of 1–3 weeks (Menzies and Gold, 1971). However, long-term changes in the expression of PGC-1α and TFAM are sufficient to drive changes in total mitochondrial content (Wu et al., 1999) and the abundance and transcription of mtDNA, respectively (Larsson et al., 1998). Moreover, evidence from human studies suggest that a 38% decrease in mitochondrial density may be enough to predispose humans to diabetes (Morino et al., 2006). Therefore, it will be extremely interesting to examine the consequences of long-term rapamycin treatment on mitochondrial function *in vivo*.

Interestingly, a third potential mechanism by which rapamycin could cause insulin resistance in skeletal muscle was recently described by Blattler et al. (2012), who showed that 2 weeks of rapamycin treatment decreased expression of YY1 target genes, including upstream components of the insulin signaling cascade. This mechanism is unlikely to be important in our C2C12 myotube model because YY1 interacts with mTORC1, and our shRNA experiments implicate mTORC2 inhibition as the cause of insulin resistance. In addition, the time frame for our experiments is much shorter, allowing less time for changes in protein level. For example, the expression of IRS1, one of the YY1 targets studied by Blattler et al. (2012), is unchanged in myotubes after 64 h of rapamycin treatment (**Figure 4D**). However, this mechanism may play a role in the longer-term effects of rapamycin on insulin sensitivity in skeletal muscle *in vivo*.

It is also intriguing to speculate that rapamycin may have effects that are independent from the mTOR complexes, in cells and *in vivo*. Rapamycin binds directly to FKBP12 and FKBP12.6, and inhibits mTORC1 via a drug-induced interaction (Sabatini et al., 1994). FKBP12 proteins have mTORC1-independent functions in the regulation of calcium channels that can lead to cardiac (Xin et al., 2002) and neuronal (Gant et al., 2011) phenotypes, and FKBP12.6 null mice exhibit strain-dependent alterations in insulin secretion (Noguchi et al., 2008; Chen et al., 2010). Therefore, the *in vivo* consequences of rapamycin treatment may include effects that go beyond the canonical inhibition of mTORC1 and the disruption of mTORC2.

Rapamycin is the only drug that has been proven to extend the maximum lifespan of a mammalian species in a rigorous, multi-center trial using lean, healthy animals that are not predisposed to any specific disease (Harrison et al., 2009). In contrast, many other molecules that improve measures of health and produce benefits that are thought to be consistent with longevity have failed to achieve an extension of maximum lifespan (e.g., Pearson et al., 2008; Miller et al., 2010; Smith et al., 2010). Although rapamycin is in clinical use, its detrimental side effects on lung health, serum lipid profiles, and immune function, as well as a possible increased risk of diabetes, are likely to preclude its use in healthy humans (Teutonico et al., 2005; Larsen et al., 2006; Stallone et al., 2009). Therefore, there is an urgent need and opportunity to understand how rapamycin works at the molecular level. The present results improve our understanding of how rapamycin influences mitochondria and insulin signaling, and lay the groundwork for future studies in cells and *in vivo*.

## Conflict of Interest Statement

The authors declare that the research was conducted in the absence of any commercial or financial relationships that could be construed as a potential conflict of interest.
